# A novel polyclonal antibody-based sandwich ELISA for detection of *Plasmodium vivax* developed from two lactate dehydrogenase protein segments

**DOI:** 10.1186/1471-2334-14-49

**Published:** 2014-01-30

**Authors:** Luciana Pereira Sousa, Luis André Morais Mariuba, Rudson Jesus Holanda, João Paulo Pimentel, Maria Edilene Martins Almeida, Yury Oliveira Chaves, Davi Borges, Emerson Lima, James Lee Crainey, Patricia Puccinelli Orlandi, Marcus Vinicius Lacerda, Paulo Afonso Nogueira

**Affiliations:** 1 ILMD, Instituto Leônidas and Maria Deane, 476, Teresina Street, 69057-070 Manaus, AM, Brazil; 2 CEPEM, Centro de Pesquisa Medicina Tropical, 4,5 km 364 Road, 78900-970 Porto Velho, RO, Brazil; 3 UFAM, Universidade Federal do Amazonas, 330, Alexandre Amorim Street, Aparecida, Manaus, AM, Brazil; 4 FMT-HDV, Fundação de Medicina Tropical Doutor Heitor Vieira Dourado, 25, Pedro Teixera Avenue, Dom Pedro, Manaus, AM, Brazil

**Keywords:** Malaria diagnosis, Lactate dehydrogenase, Recombinant protein, ELISA

## Abstract

**Background:**

Immunoassays for Plasmodium detection are, presently, most frequently based on monoclonal antibodies (MAbs); Polyclonal antibodies (PAbs), which are cheaper to develop and manufacture, are much less frequently used. In the present study we describe a sandwich ELISA assay which is capable of detecting *P. vivax* Lactate Dehydrogenase (LDH) in clinical blood samples, without cross reacting with those infected with *P. falciparum*.

**Methods:**

Two recombinant proteins were produced from different regions of the *P. vivax* LDH gene. Two sandwich ELISA assay were then designed: One which uses mouse anti-LDH 1-43aa PAbs as primary antibodies (“Test 1”) and another which uses anti-LDH 35-305aa PAbs (“Test 2”) as the primary antibodies. Rabbit anti-LDH 1-43aa PAbs were used as capture antibodies in both ELISA assays. Blood samples taken from *P. vivax* and *P. falciparum* infected patients (confirmed by light microscopy) were analysed using both tests.

**Results:**

“Test 2” performed better at detecting microscopy-positive blood samples when compared to “Test 1”, identifying 131 of 154 positive samples (85%); 85 positives (55%) were identified using “test 1”. “Test 1” produced one false positive sample (from the 20 malaria-free control) blood samples; “test 2” produced none. Kappa coefficient analysis of the results produced a value of 0.267 when microscope-positive blood smears were compared with “test 1”, but 0.734 when microscope-positive blood smears were compared with the results from “test 2”. Positive predictive value (PPV) and negative predictive value (NPV) were observed to be 98% and 22% respectively, for “Test 1”, and 99% and 45%, for “test 2”. No cross reactivity was detected with *P. falciparum* positive blood samples (n = 15) with either test assay.

**Conclusion:**

Both tests detected *P. vivax* infected blood and showed no evidence of cross-reacting with *P. falciparum.* Further studies will need to be conducted to establish the full potential of this technique for malaria diagnostics. As well as representing a promising new cost-effective novel technique for *P. vivax* diagnosis and research, the method for developing this assay also highlights the potential for PAb-based strategies for diagnostics in general.

## Background

Malaria is one of the most widespread infectious diseases, carrying with it an enormous cost in human suffering and economic hardship. In 2010 there were an estimated 216 million cases of the disease [1] and over half a million deaths attributed to it [2]. Despite great advancements in many areas of malaria research, light microscopy-based methods of parasite detection, which were first developed over a century ago, still remain as the gold standard and the most commonly used way of detecting malaria parasites in blood samples [3].

Although this technique is cost-effective and permits quantitative and qualitative parasite detection, it has some quite serious limitations. As well as requiring specialist equipment and staff training, light microscopy also needs basic infrastructural support and is unable to detect very low levels of parasitemia [4,5]. As a result of these limitations, prevalence levels can be underestimated and diagnostic errors can be made, which can lead to suboptimal malaria treatment regimens [6,7].

PCR-based malaria detection offers a greater level of sensitivity than light microscopy [8]; however, it too also requires specific apparatus, technical experience and even more complex and expensive infrastructural support (especially if the technique is required to have a quantitative dimension) [9]. Thus while PCR has helped to illuminate the limitations of light microscopy-based malaria detection, it is still not a major part of routine malaria parasite detection, especially in remote areas where malaria can be prevalent.

Immunological assays which use antibodies to detect parasite molecules offer a fundamentally different approach to both PCR and light microscopy. Although these assays can also suffer from sensitivity issues, they have a far greater potential for adaption to infrastructure-free settings than the other existent approaches. For this reason Immunological assays have become basis of most commercial diagnostic test kits, with most interest focused on the use of monoclonal antibodies (MAbs) [10,11]. Almost all of the malaria rapid diagnostic tests (RDTs) that are presently available on the commercial market make use of MAbs to detect the presence of parasite proteins. Although such MAb-based methods offer a potentially powerful infrastructure-free way of diagnosing the presence of malaria parasites and are increasingly being used for this purpose, RDTs are still only used for a small fraction of the hundreds of thousands of malaria diagnoses made annually [1]. Part of the reason for this is that RDT test kits are still too expensive for routine usage in developing counties where most malaria occurs [12] and part of the reason is that RDTs can be unreliable and often recommend that diagnosed results are validated with light microscopy anyway [13-19].

While MAb-based ELISA assays are generally more sensitive than MAb-based rapid diagnostic tests (RDTs) and they can offer certain advantages over PCR and light microscopy for malaria diagnosis, the infrastructure requirements for their use in ELISA assays are similar to those needed for PCR and light microscopy diagnoses. In contrast to MAbs, polyclonal-antibodies (PAbs) are far simpler and cheaper to develop and manufacture [20] and therefore offer the potential to lower the cost of both RDTs and laboratory-based ELISA assays for malaria diagnosis and, by doing so, potentially broaden the appeal of Immunoassays in both laboratory and field settings. PAb–based immunoassays can, however, sometimes be more sensitive to false positives, than MAb-based assays [21], which is a problem that this paper has attempted to address.

In this paper we describe the development of a novel polyclonal based technique for the detection of the Malaria parasite *P. vivax*. The technique developed has avoided cross-reactivity issues by using two polyclonal antibody sets in concert: one directed to a short portion of the n-terminal region of the *P. vivax* lactate dehydrogenase (*Pv*LDH) and one to a larger over-lapping portion of the same protein.

## Methods

### Blood sample collection from patients infected with *Plasmodium vivax*

Red blood cells (RBC) of 154 patients infected with *Plasmodium vivax* were collected between March of 2010 and February of 2011. RBC of 15 patients infected with *P. falciparum* were also collected. *Plasmodium vivax* and *P. falciparum* were confirmed with light microscopy. Secondary laboratory confirmation of *P. falciparum* blood infections was obtained by ELISA using an anti-HRP2 (Histidine rich protein 2) *P. falciparum* specific assay, described previously. A control group was formed with twenty blood samples taken from healthy individuals who were not thought to have been exposed to malaria for more than 6 month. Following collection, all samples were centrifuged; serum and erythrocytes were then separated and stored at -20°C until their use in the ELISA assays, described below.

### Recombinant protein production and quality assessment

**Figure 1:**
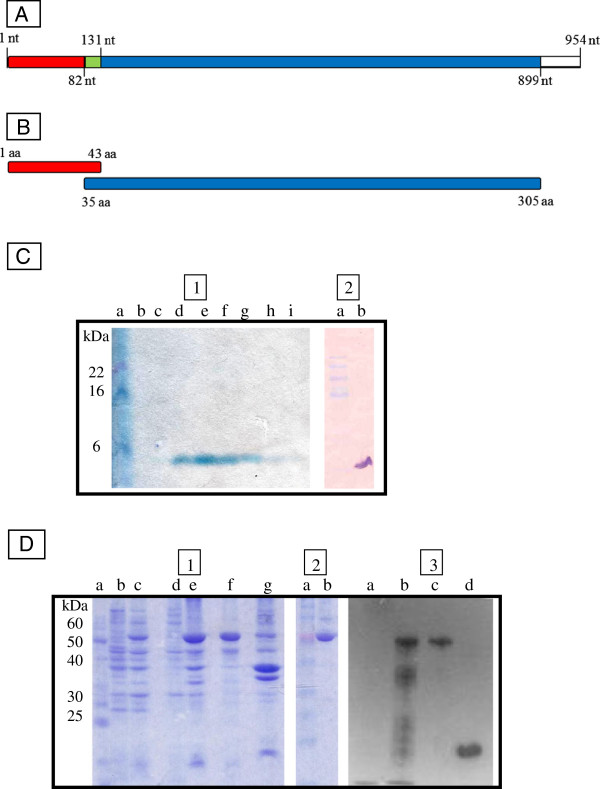
**Recombinant protein production for pvLDH epitope preparations. A)** Shows the pvLDH gene sequence targeted for “p*v*LDH 1-43aa” protein production; PCR primers were used to amplify nucleotide positions 1 to 131 (shown in red and green). For “p*v*LDH 35-305aa” protein production, PCR primers were designed to amplify nucleotide positions 82 to 899 (shown in green and blue). **B)** Shows the corresponding protein segments “p*v*LDH 1-43aa” and “p*v*LDH 35-305aa” that were subsequently cloned and expressed. **C)** Shows the results from a SDS page gel and an immunoblot that were used to confirm the successful expression and isolation of a recombinant proteins of the size expected of p*v*LDH 1-43aa protein segments. **D)** Shows the results from a western blot confirming the successful expression and isolation of a recombinant protein of a size expected for p*v*LDH 35-305aa recombinant protein segment. NT = Nucleotide; AA = Aminoacids.

As a first step in the production of polyclonal antibodies for detection of native LDH from *P. vivax* (pvLDHn), two recombinant proteins were designed (see Figures 1A and B). The first protein (*pv*LDH1-43) was designed to contain the amino acid residues corresponding to positions 1° to 43° of the *pv*LDHn protein (i.e. an extension of the region used successfully by Piper et al. (1999) [10] in a similar MAb immunoassay and second protein (*pv*LDH35-305) was designed to contain the amino acid residues from positions 35° to 305° of the *pv*LDHn protein (aiming the production of antibodies capable to interact with a large area of antigen target). Molecular mass predictions for each of the two designed proteins were made using the program Protparam (Expasy).

For *Plasmodium vivax* genomic DNA extraction, 100 μl of erythrocytes’ sediment was treated with 1% saponin in Salt phosphate buffer for 20 minutes. After centrifugation the pellet was resuspended in distilled water and treated with lysis buffer (40 mM Tris, pH 8; 80 mM EDTA; 2%SDS; 0,1 mg/ml of K-proteinase) for 16 hours. Distilled water was added to make-up each preparation to a 500 μl volume; five hundred microlitres of phenol were then added to the preparation and the resultant 1 ml solution was homogenized and centrifuged at 12000 rpm for 5 minutes. After centrifugation, the aqueous phase was collected and homogenized with chloroform; 250 μl of the aqueous phase of this preparation was then added to 45 μl of 3 M of sodium acetate. Genomic DNA was then precipitated with 100% ethanol. DNA was then pelleted with centrifugation and then washed with 70% ethanol and centrifugation.

For production of pvLDH1-43, two oligonucleotide primers were used to amplify the targeted region for cloning: the forward primer was 5´ ggatccATGACGCCGAAACCCAAAATTGT 3´ and reverse primer was 5´ gaattcTTTCCTTGGGGCCATGTTTTT 3´. The reaction mixture used for PCR amplification was prepared containing: 1X Taq DNA polymerase buffer, 2.25 nM MgCl2, 0,125 mM dNTP (Invitrogen), 0.6 pMol of each oligonucleotide primer, around 100 pg genomic DNA and 1 unit of Taq polymerase enzyme (Invitrogen) in a final volume of 50 μL. Sterile distilled water was used to make a final reaction volume of 25 μl. PCR conditions were as follows: One initial denaturing step at 94°C for 5 min; followed by 30 cycles of denaturing at 94°C for 1 minute, annealing at 69°C for 30 seconds and extending at 72°C for 1 minute; and a final extension step at 72°C for 10 minutes. The resultant PCR product was purified using a Gel Extract kit (Qiagen) and then cloned using a commercially purchased vector (pGEM-T Easy plasmid, Promega) and competent *E. coli* cells. A plasmid preparation of pGEM-pvLDH 1-43aa was then made using a QIAGEN miniprep kit and digested with the restriction enzymes *Bam*H1 and *Eco*R1 (Invitrogen). The digested plasmid insert was then isolated by gel extraction (using a Qiagen gel extraction kit) and then directional cloned into an expression vector using pRSET A (Invitrogen) and *E. coli* competent cells. Successful cloning of the targeted gene sequence was then confirmed by Sanger sequencing the pRSETA-pvLDH 1-43aa insert in two directions using vector and insert primers and an Abi 3100 genetic analyzer and recommended reagents and protocols (Applied Biosystems).

After sequence integrity confirmation, purified plasmid pRSETA-*pv*LD 1-43aa was used to transform competent *E. coli* BL21 (DE3) pLysS cells for expression. Transformed colony isolates were incubated in a 200 ml LB ampicillin culture at 37°C, with constant agitation until the cell density provided an OD (optical density) reading of 0.8. At this point, induction of the gene expression was initiated with isopropyl β-D-1-thiogalactopyranoside (IPTG) (at a final working concentration of 1 mM). Following induction, the culture was incubated, as before, for a further 3 hours. The cell suspension was subsequently centrifuged at 14 000 rpm, at 4°C, for 15 minutes and the pelleted cells were resuspended in buffer 2 (20 nM Tris, pH 7.9; 0.5 M NaCl; 10% glycerol; 1 mM phenylmethanesulfonylfluoride), and left, rocking, for 30 minutes. Recombinant proteins, contained in the resultant soluble fraction were decanted from cell debris after centrifugation at 14 000 rpm at 25°C for 15 minutes and were then isolated using Ni-NTA columns (QIAGEN) for purification of recombinant protein under denaturing conditions, following the manufacture’s recommendations. SDS-PAGE gels were used to monitor protein expression and purification, and an anti-HisG antibody (Invitrogen) immunoblot (with Western Breeze kit [Invitrogen]) was used to ensure the presence of polyhistidine tag in the recombinant protein.

For production of pvLDH35-305, the forward primer used was 5´ GGATCCATGACGTAGTGAAAA 3´ and reverse primer used was 5´ GAATTCAACTGCCTCGTCG 3´. The reaction mixture used for gene amplification was prepared containing 1X Taq DNA polymerase buffer, with 3 mM MgCl2, 0.15 mM dNTPs, 15 pMol of each oligonucleotide primer, 100 pg genomic DNA and 1 unit of Taq polymerase enzyme (Invitrogen) in a final volume of 50 μL. The PCR conditions used for amplification were as follows: one initial denaturing step at 94°C for 5 minutes; followed by 30 cycles of a denaturing step at 94°C for 1 minute followed by an annealing at 62°C for 1 minute and an extension step at 72°C for 1 minute; and then finally a 72°C final extension step lasting 5 minutes. The resultant PCR product was purified and cloned into a protein expression vector The approach used to create and assay protein production from pGEX3X-*pv*LDH 35-305aa was very similar to that used to create from pRSETA-*pv*LDH 1-43aa. Purification of GST tagged protein was conducted with a glutathione sepharose kit following the manufacture’s instructions (Amershan).

### Polyclonal antibodies attainment and purification

Two groups of three mice and one rabbit were immunized separately with pvLDH1-43 protein serum. Mice and rabbits were stimulated with 50 μg and 500 μg of recombinant protein, respectively. Three inoculations were carried-out in both kinds of animal with intervals of 15 days, using complete freund’s adjuvant in first inoculum and incomplete freund’s adjuvant in the others. Indirect ELISA using animal sera were used to monitor humoral response. The most responsive animals, total serum was obtained and IgG was purified using Protein A sepharose (Amershan) [22]. Animals were bled and serum purified in polyclonal antibody preparations with concentrations of 1 μg/μL and 6 μg/μL from mice and rabbits, respectively. Purified pvLDH35-305 was inoculated just in mice for this study.

### ELISAs for antigen detection

**Figure 2:**
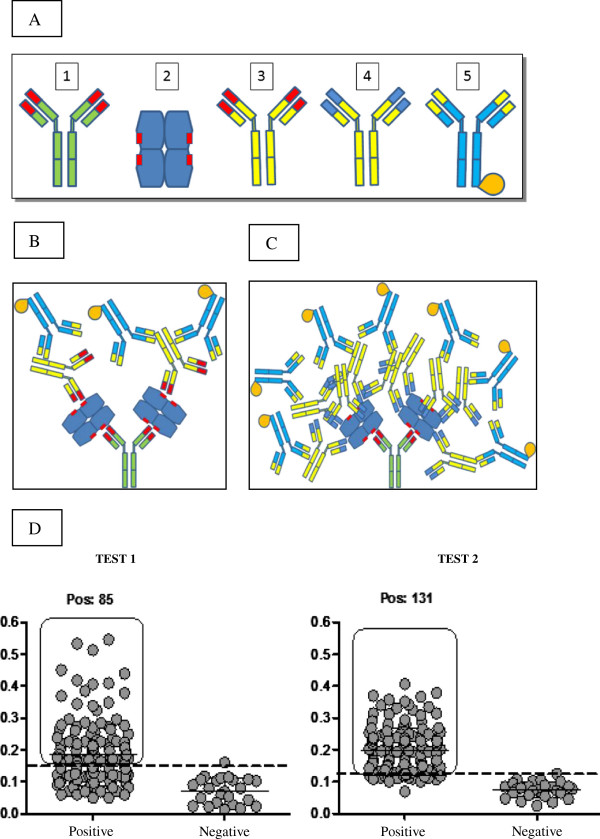
**pvLDH PAb Sandwich ELISA assays. A)** Shows the systems components labeled: 1) Rabbit antibody anti-p*v*LDH 1-43 aa; 2) Native LDH (draw based in tetramerical structure described by Chaikuad (2005); 3) Mouse antibody anti- p*v*LDH 1- 43 aa; 4) Mouse antibody anti-p*v*LDH 35-305aa; 5) Goat secundary antibody anti-mouse IgG conjugated to HRP. **B)** Shows a graphic representation of “test 1”. **C)** Shows a graphic representation of “test 2”. **D)** Shows two dot plots illustrating performance of the two PAb assays tests described in main text. Values indicated on the Y-axis are for optical density measurements. Individual data points are represented with spots. In both test data sets, points from negative control blood samples are displayed on the right hand side of the plots and those from the microscope slide positive samples are shown on the left. Solid horizontal lines represent test mean values, dashed lines indicate test cut-off points.

Sandwich ELISAs for LDH *P. vivax* and *P. falciparum* detection were done using rabbit and mouse purified IgGs. Two systems were tested: a two-site polyclonal antibody sandwich ELISA using just anti-*pv*LDH 1-43aa (coating plates with rabbit antibody and mouse antibody as primary, (“test 1” Figure 2B), and a two site polyclonal antibody sandwich ELISA using anti-*pv*LDH 1-43aa (for coating) and anti-*pv*LDH 35-305aa (as primary) (“test 2”, Figure 2C).

Briefly, ELISA plates were coated with 50 μl of rabbit polyclonal antibodies anti-*pv*LDH 1-43aa at a concentration of 4 μg ⁄mL in sodium bicarbonate buffer (pH 9.6). The coated plates were incubated at 4°C overnight. After blocking at 37°C with phosphate buffer containing 5% skimmed milk (w⁄v) for 2 h, plates were washed with phosphate buffer. Test sample (12,5 μL of erythrocyte concentrate plus 37,5 μL of 0,1% Triton x-100) were then added in duplicates into the plates and incubated for 1 h at 37°C. After the washing step with the phosphate buffer, primary mouse antibody at a concentration of 16 μg/ml was added. Bound antibodies were detected with HRP (Horseradish peroxidase) conjugated goat anti-mouse IgG antibodies (adsorbed to rabbit antibodies, KPL), and reveled using TMB (0.1 mg/ml final concentration), 0,04% f.c. H_2_O_2_, in phosphate-citrate buffer.

### Alignment and linear B-cell epitopes analyses

Residues 1-43aa of lactate dehydrogenase from *P. vivax* (AEP83563.1)*, P. ovale* (AAS77571.1)*, P. malariae* (AAS77572.1)*, P. falciparum* (ABH03417.1)*, P. berguie* (AY437808.1)*, P. knowles* (JF958130.1) and *P. yoelli* (XP_724101.1) were downloaded from the NCBI protein databases and then edited manually before being aligned using Clustal W2. Detection of linear B-cell epitopes was done using Bepiprep 1.0 server (Larsen & Nielsen, 2006) and a threshold value of 0.2.

### Data management and statistical analysis

Data was recorded on registered forms and entered into a Microsoft Excel spreadsheet (Microsoft Corporation). Cut-offs were calculated using the mean optical density readings from negative sample plus twice the standard deviation of negative samples. Calculation of specificity, sensitivity, positive predictive and negative predictive values followed procedures detailed in [23]. Agreement for results of microscopy and ELISA, and between the two ELISA methods was calculated with kappa values. A kappa value between 0.6 and 0.8 was considered a good agreement, higher than 0.8 was considered as excellent [24].

### Ethical review

The study was approved by the Brazilian Animal Ethical Committee (CEEA-UFAM 005/2010) and the Human Research Ethical Committee of Amazonas Federal University (CAAE 3640.0.000.115-07). All patients signed a statement of consent.

## Results and discussion

Most Malaria immunological assays target one of three malaria proteins: Histidine rich protein 2 (HRP2), aldolase or lactate dehydrogenase (LDH), all of which are known to be abundant and detectable in blood serum. Studies on LDH, however, have shown that blood concentrations of the LDH proteins correlate strongly with parasite blood-levels and thus assays targeting this protein can have a quantitative dimension. Although the development of a quantitative assay what not an initial goal of this work, and outside the scope of the work presented here, this feature contributed to its selection as a target for this work.

DNA sequence analysis confirmed that the two LDH gene segments that were targeted for this study (*pv*LDH 1-43aa and *pv*LDH 35-305aa) were successfully cloned into plasmid expression vectors. The successful expression of recombinant proteins from their vectors was confirmed with Western blot (see Figures 1C and D). Initial sandwich ELISA assays carried out with the PAbs generated from the *pv*LDH 35-305aa showed unacceptably high levels of cross-reactivity (data not shown) in negative samples, presumably caused by the presence of native human LDH in blood samples. The pvLDH 1-43aa PAbs developed here were specifically designed to combat this issue. Rabbit and mice PAbs directed to *pv*LDH 1-43aa, when used in combination as capture and primary antibodies respectively in “test 1” (see Figure 2B and C), showed unacceptably low level of parasite detection (only 85 of 154 [55%] microscope positive samples tested positive with this test), although they did not show much evidence of cross reaction: just one of the malaria-free control group blood samples (1/20) tested positive for the parasite with this assay (see Figure 2D). One possible explanation for this observation is that there is epitope binding competition between the capture and the primary antibodies used in this assay. Even though the tetrameric structure of the antigen [25] might be expected to limit the impact of such binding competition, such competition affects have been reported elsewhere [26].

The second sandwich ELISA experiment tested here was designed to harness the strengths of both assays and evade the weaknesses. In this assay (see test 2 in Figure 2D) thus anti-*pv*LDH *pv*LDH 1-43aa PAbs were used as the initial capture antibodies and the anti-*pv*LDH 35-305aa PAbs were used as primary antibodies. This test showed higher levels of parasite detection than were observed for “test 1”: A total of 131 (131/154; 85%) of the microscopy positive samples tested positive in this assay and the assay did not generate any false positives (see Figure 2D). These results show that the tested *pv*LDH 35-305aa PAbs are more affective primary antibody complements to *pv*LDH 1-43aa PAbs primary antibodies than the *pv*LDH 1-43aa PAbs are. Presumably this is because the 35-305aa PAbs bind more affectively to *pv*LDH 1-43aa-MAb-bound native *pv*LDH proteins than 1-43aa PAbs do, perhaps because of reduced competition. The fact that no cross reactivity was observed with this assay is likely to be a consequence of the removal of human LDH protein during the ELISA wash steps that preceded the introduction of the secondary anti-35-305aa PAbs.

**Figure 3:**
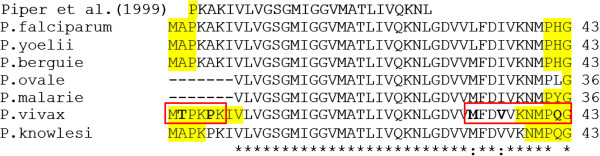
**Alignment of residue 1-43aa from diferent *Plasmodium *species.** Highlighted amino acids was the detected ones in Bepiprep (threshold = 0.2). Bold letters and red box represents the diference between *P. vivax* and *P. falciparum* at this region.

**Figure 4:**
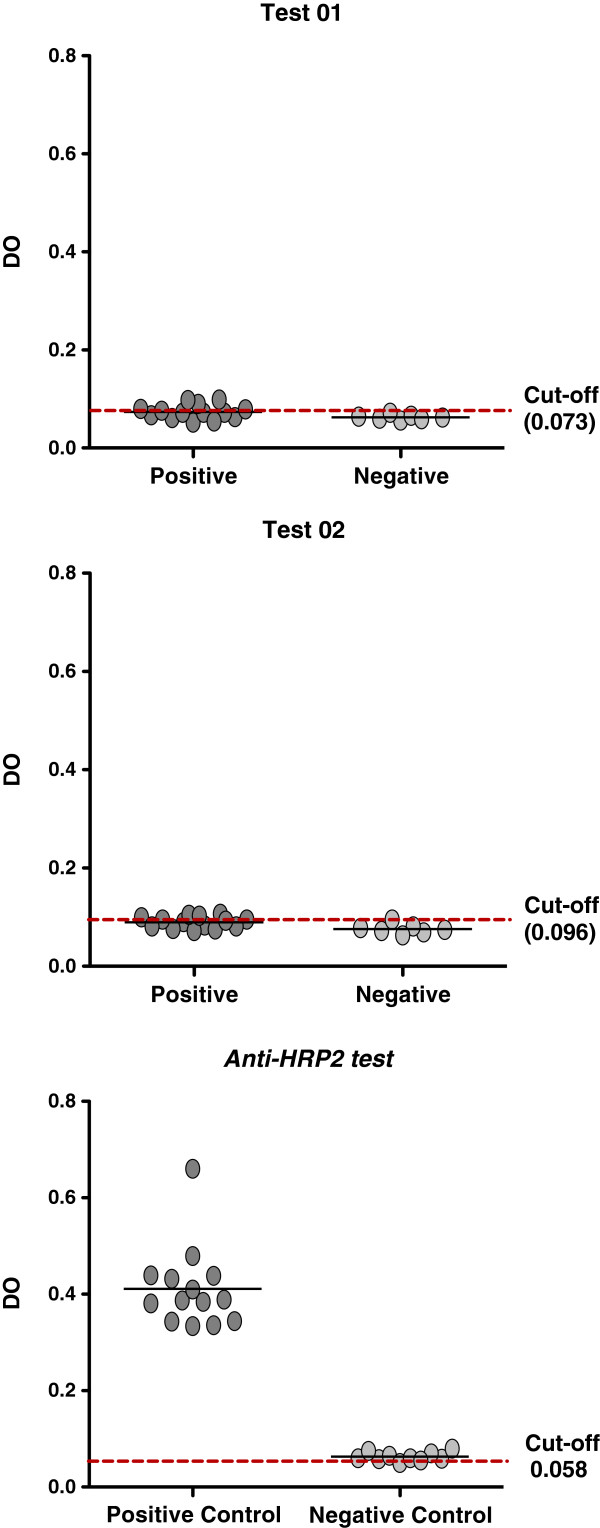
**ELISA results of *P. falciparum *infected blood samples.** Test 1 and 2 did not presented positivite results. Control assay using anti-HRP2 antibodies detected all positive samples.

Surprisingly, our polyclonal antibodies were not able to detect parasites in microscope positive (and HRP2 ELISA confirmed) *P. falciparum* infected blood samples using either the “test 1” or “test 2” methodology (see Figure 3). Figure 4 shows our pvLDH 1-43aa target protein aligned to six equivalent segments from other plasmodium species and shows a region of the protein which has already been used in the development in a pan-*Plasmodium* sandwich ELISA. Although the apparent specificity of our assays maybe limited to the Amazonian derived *P. falciparum* samples we have tested, the low levels of between-species diversity, which can be seen in Figure 4, occur in protein regions our bepiprep analysis has predicted are most likely to be immunogenic and therefore our assay may retain this specificity for a broader range of *P. falciparum* strains.

The combination dual-LDH PAb sandwich ELISA assay described here in test 2 thus represents a potentially powerful low cost tool, which could form the basis of a variety of immunological assays, for research and clinical diagnosis and parasite load monitoring. Using microscopy diagnosed slides as the gold standard, the positive predictive values (PPV) and negative predictive values (NPV) calculated for these tests were 98% and 22% (for “test 1”) 99% and 45%, for “test 2”. Kappa coefficient analyses of the results gave a 0.267 value when blood smears were compared to “test 1”; a 0.734 value when blood smears were compared to the results from test “test 2” and a value of 0.367 when both test 1 and 2 results were compared with each other. Although the potential for this assay to detect submicroscopic levels of parasitemia has not yet been examined, this assay compares very favorably when compared to PCR for detecting microscope positive blood samples [4]. Similarly, when the results from “test 2” are compared with the results obtained with commercially available MAb-based immunoassays its performance can be seen to comparable [27]. In addition to this, as the blood samples tested in this assay were all stored frozen prior to use it might also be that the sensitivity of the test can be improved with fresh blood samples as Kifude and colleagues (2008) [28] showed that HRP-2 signal detection reduces after samples are subjected to freeze and thaw cycles.

## Conclusion

In conclusion, the novel LDH targeting PAb-based sandwich ELISA “test 2” described here detects *P. vivax* microscopy-positive blood samples with an efficiency similar to what has been observed with MAb-based RDT kits. Further studies will need to be conducted to establish if the technique can be adapted to detect submicroscopic parasitic infections and/or if it can perform better with fresh blood samples and/or if it can be adapted for a RDT. As well as representing a promising new cost-effective novel technique for *P. vivax* diagnosis and research, the method for developing this assay also highlights the potential for PAb-based strategies for diagnostics in general. It is thus suggested here that the both dual antibody LDH sandwich ELISA described here and the technique used to generate it might stimulate further research in this field.

## Competing interests

The authors declare that they have no competing interests.

## Authors’ contributions

LAMN and PAN conceived, designed the study and drafted the manuscript. LPS, RJH, MEMA, YOC, DB all made substantial contributions to the acquisition of the data presented in this manuscript. EL, PPO, MVL, PAN also all made important contributions to the data contained in this article and were involved in the studies design and execution. JLC made a substantial contribution to the article’s drafting and revision for publication. All authors read and approved the final manuscript.

## Pre-publication history

The pre-publication history for this paper can be accessed here:

http://www.biomedcentral.com/1471-2334/14/49/prepub
